# The Effects of Grain Boundaries on the Current Transport Properties in YBCO-Coated Conductors

**DOI:** 10.1186/s11671-015-1124-8

**Published:** 2015-10-26

**Authors:** Chao Yang, Yudong Xia, Yan Xue, Fei Zhang, Bowan Tao, Jie Xiong

**Affiliations:** State Key Laboratory of Electronic Thin Films and Integrated Devices, University of Electronic Science and Technology of China, Chengdu, 610054 China

**Keywords:** YBa_2_Cu_3_O_7-δ_ (YBCO), Grain boundary (GB), Current carry capacity, Misorientation angles

## Abstract

We report a detailed study of the grain orientations and grain boundary (GB) networks in Y_2_O_3_ films grown on Ni-5 at.%W substrates. Electron back scatter diffraction (EBSD) exhibited different GB misorientation angle distributions, strongly decided by Y_2_O_3_ films with different textures. The subsequent yttria-stabilized zirconia (YSZ) barrier and CeO_2_ cap layer were deposited on Y_2_O_3_ layers by radio frequency sputtering, and YBa_2_Cu_3_O_7-δ_ (YBCO) films were deposited by pulsed laser deposition. For explicating the effects of the grain boundaries on the current carry capacity of YBCO films, a percolation model was proposed to calculate the critical current density (*J*_c_) which depended on different GB misorientation angle distributions. The significantly higher *J*_c_ for the sample with sharper texture is believed to be attributed to improved GB misorientation angle distributions.

## Background

In the past decades, YBa_2_Cu_3_O_7-δ_ (YBCO) thin films deposited on single crystal achieved a high critical current density (*J*_c_) in the range of 1~10 MA/cm^2^ at 77 K, self field [1]. Tremendous efforts have been devoted to fabricate high performance YBCO thin films on flexible metal tapes as promising high temperature superconductor (HTS) wires for power application or high field magnets [2, 3]. However, YBCO deposited on polycrystalline substrates exhibited high angle grain boundaries (GBs) between the grains, which acted as Josephson coupled weak-links behavior, drastically reducing the current carry capacity, i.e., *J*_c_ smaller than 1 MA/cm^2^ [4, 5]. Dimos et al. [6] reported the dependence of *J*_c_ on the misorientation angles. For GBs with low angles (*θ* < 5°), the critical current densities decreased slightly when the bicrystal misorientation angles *θ* increased, and the behavior was similar to that exhibited in single grains. However, with increasing misorientation angle *θ* in bicrystals at a range of 15°~20°, *J*_c_ decreased very rapidly. Horide et al. [7] reported that the current transport conformed with Josephson junction behavior for a large angle *θ.* For depositing well-aligned coated conductors, two techniques were successfully explored to prepare the biaxial texture. The first was ion beam-assisted deposition (IBAD) to deposit single-crystal-like films on polycrystalline metal substrates [8, 9]. Whereas the second, named rolling-assisted biaxially textured substrate (RABiTS), was used for thermomechanical processing to achieve biaxially texture Ni-alloy tapes directly [10, 11].

In RABiTS technology, rolling and annealing of the substrate result in the formation of a sharp {100} <100> cube texture. The typical textures of the tapes are 5°~7° decided by X-ray *ω*-scan (out-of-plane) and *Φ*-scan (in-plane) full-width at half-maximum (FWHM) values. Unfortunately, inherent grain boundary networks from annealing of metal recrystallization would seriously deteriorate the superconductivity. The range of total GB misorientation angles (*θ*) in RABiTS tapes is from 1° to 10° [12]. And the grain boundary networks in the RABiTS tapes are transferred through buffer layers to YBCO layer, which has been characterized by transmission electron microscopy (TEM) and electron backscatter diffraction (EBSD) [13]. According to the results on RABiTS process, the intra-grain *J*_c_ values had similarity with the *J*_c_ values in single crystal substrates [14]. The significant reduce of *J*_c_ values occurred when the current went across the large angle grain boundaries. With the *θ* increasing to 6°, the *J*_c_ values of YBCO films dropped significantly in the inter-grains [14, 15], demonstrating strong influence of the grain boundary networks on the current transport in YBCO films.

At present, physical vapor deposition (PVD) and trifluoroacetate precursor metalorganic deposition (MOD) have been successfully applied to fabricate YBCO-coated conductors. For PVD process, such as pulsed laser deposition (PLD) [16] and evaporation [17], the YBCO film exhibited a perfect epitaxy on the buffer layers, and the out-of-plane and in-plane alignments of YBCO films were similar to the buffer layers [18, 19]. It had been shown by EBSD that grain boundary networks of YBCO films totally copied the grain boundary networks of buffer layers. For the MOD process, the grain boundary networks were not only transferred from the buffer layer but also meandered in the YBCO films [20]. The meandering of the GBs in MOD resulted in a significant reduction in the GB misorientation angles [15]. Furthermore, the meandering of GBs was also found in the PVD-BaF_2_ process, which was promising technology to achieve high *J*_c_ with thick (2~3 μm) YBCO films [19]. For achieving high current carry capability of the traditional PVD process, the templates with small GB misorientation angles are necessary.

Of particular, it should be noted that the buffer stacks play a key role in YBCO-coated conductors. The purpose of buffer layers is to prevent counter-diffusion of metal atoms in substrate and oxygen atoms in YBCO. In addition, buffer layers provide with a continuous, smooth, chemical inert surface and good match for the growth of YBCO films. Therefore, the buffer layers must to be epitaxially grown on the RABiTS substrates, and the lattice parameters of buffer layers should have a small mismatch with the substrate. Many oxide materials, such as Y_2_O_3_ [21], CeO_2_ [22], SrTiO_3_ [23], and La_2_Zr_2_O_7_ [24], have been successfully attempted. Among them, the multi-layer architecture CeO_2_/YSZ/Y_2_O_3_ is most widely used.

Recently, we deposited CeO_2_/YSZ/Y_2_O_3_ buffer layers on the RABiTS using sputtering for coated conductors, different samples of Y_2_O_3_ buffered were characterized by X-ray powder diffraction (XRD) and EBSD, the results showed different textures and grain boundary networks. We focus on the texture and GB development of Y_2_O_3_ seed layer which affect the current carry capacity of YBCO deposited on CeO_2_/YSZ/Y_2_O_3_ buffered RABiTS substrates. And a percolation model was proposed to calculate the *J*_c_ of modeled samples.

## Methods

RABiTS Ni-5 at.%W (NiW) tapes with 10 mm width and 80 μm thickness were produced by EVICO GmbH, Germany. The FWHM values *ω*-scan and *Φ*-scan are 5.5° and 6°, respectively. The root mean square surface roughness (*R*_rms_) of the NiW tapes was less than 5 nm over 5 × 5 μm areas.

CeO_2_, YSZ, and Y_2_O_3_ were served as the cap layer, barrier layer, and seed layer, respectively. The Y_2_O_3_ seed layer was deposited by direct current (DC) magnetron reactive sputtering and the YSZ and CeO_2_ layers by radio frequency (RF) magnetron sputtering. Details of the experimental conditions are reported elsewhere [25]. Rocking curve (*ω*-scan) and *Φ*-scan were used to characterize the out-of-plane and in-plane texture with Bede D1 XRD, respectively. Spatially resolved maps of crystal orientation and GB misorientation angles were measured by electron backscattering diffraction (EBSD). The data were gathered in a hexagonal grid with a spacing of 1 μm in a region of 400 × 400 μm^2^. All data were processed by orientation image micrograph (OIM) patterns. The sample normal was parallel to the map normal. The grain boundary network maps were superimposed on the background of the grain images, and the grain misorientation angles comprise with both in-plane ([001] tilt) and out-of-plane ([100] tilt and [100] twist) misorientation.

YBCO films were deposited by PLD. In brief, a substrate temperature of 775 °C and an oxygen pressure of 200 mTorr were used for the deposition of YBCO. Critical current density (*J*_c_) measurements were performed at 77 K, using a four-probe technique and a 1 μv/cm voltage criterion [26].

## Results and Discussion

**Fig. 1:**
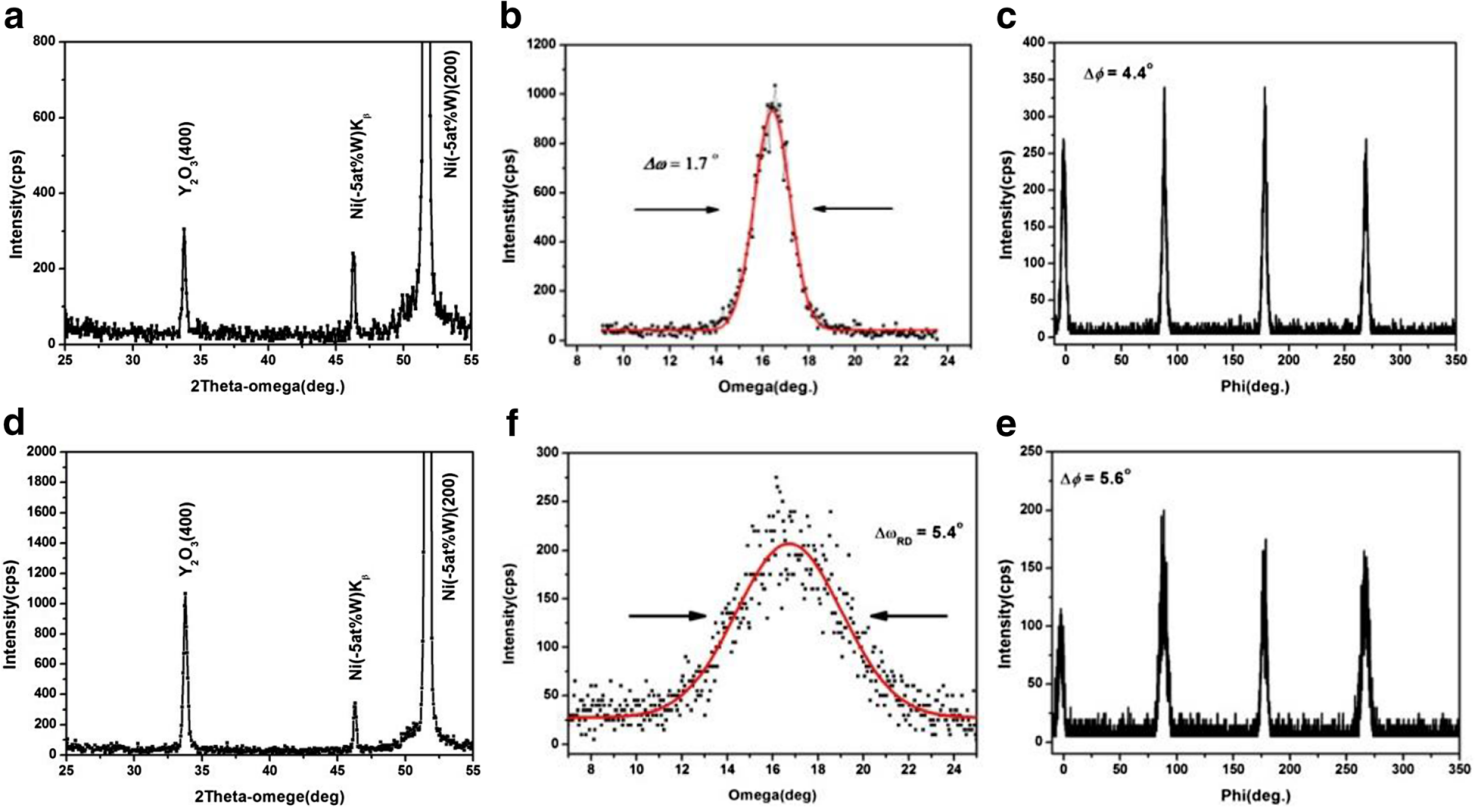
*θ*-2*θ*-scan, *ω*-scan, and *Φ*-scan of two samples determined by XRD, representative of the Y_2_O_3_ seed layer used in this work. **a**–**c** Sample 1, **d**–**e** sample 2

Figure 1 shows XRD patterns of the two samples of the Y_2_O_3_ seed layer with different textures. Both samples indicated excellent *c*-axis orientations. *θ-*2*θ* scan, *ω*-scan and *Φ*-scan of sample 1 showed in Fig. 1a–c, which exhibited a very sharp out-of-plane and in-plane texture with FWHM values were 1.7° and 4.4°, respectively. The significant improvement in both out-of-plane and in-plane alignments of about 4° and 1.5° was observed compared with the substrate. The single-crystal-like out-of-plane texture was attributed to out-of-plane titles of Y_2_O_3_ grown on the NiW template grains [27]. Figure 1e, f shows that the FWHM values for out-of-plane and in-plane textures were 5.4° and 5.6°, respectively.

**Fig. 2:**
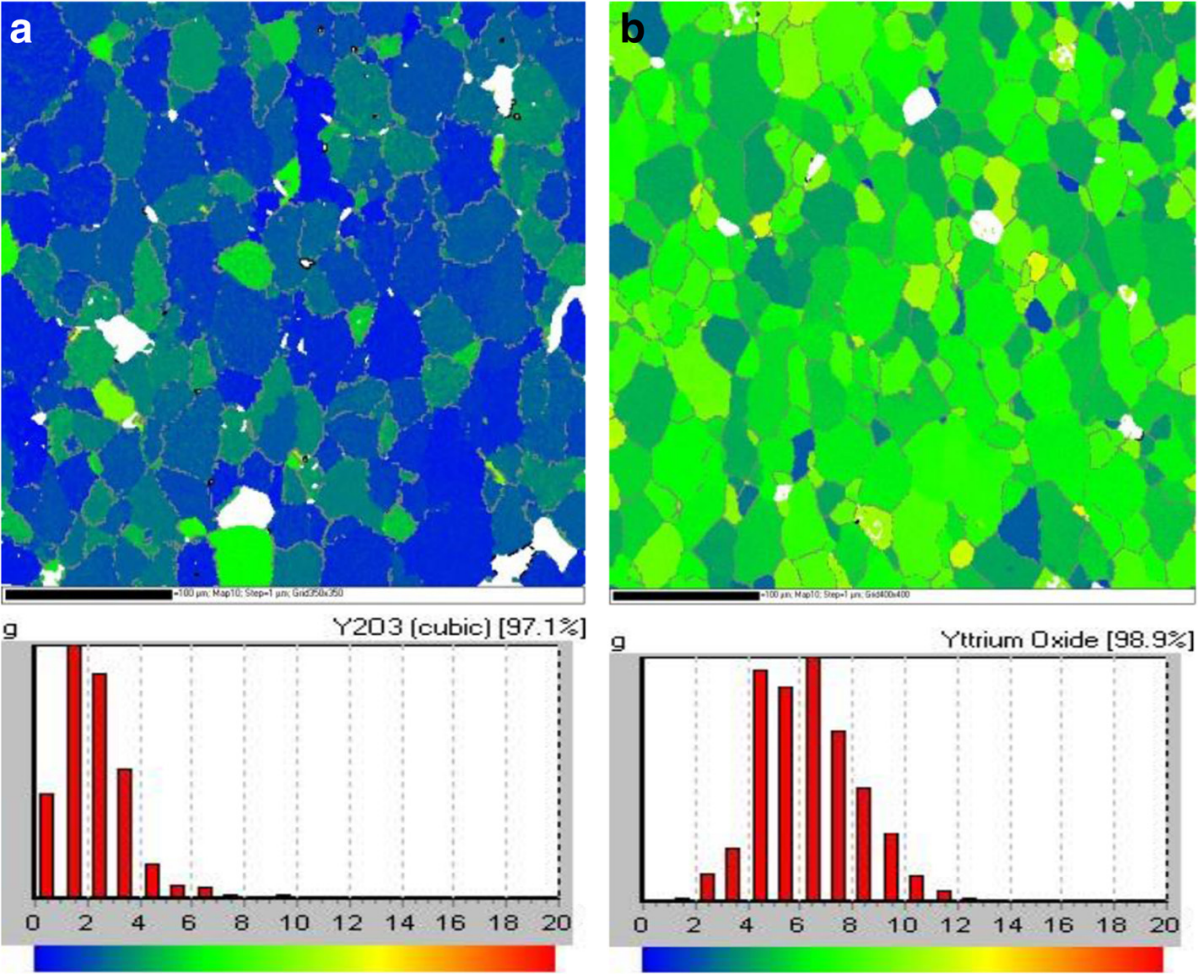
OIM patterns of Y_2_O_3_ seed layer: **a** sample 1, **b** sample 2

**Fig. 3:**
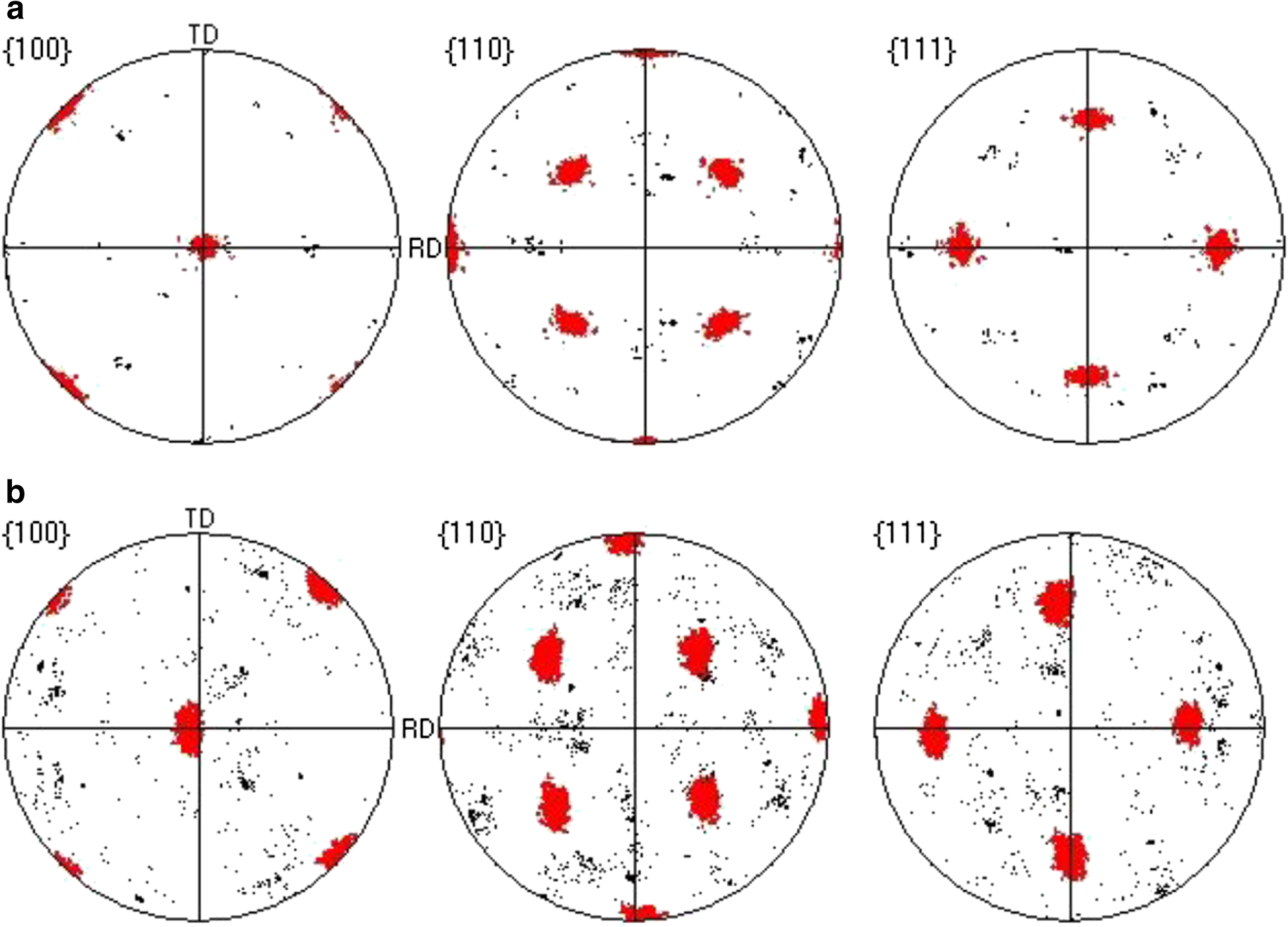
(100), (110), and (111) pole figures of Y_2_O_3_ seed layer calculated by EBSD data: **a** sample 1, **b** sample 2

Figure 2 shows EBSD OIM patterns of two sample of the Y_2_O_3_. The data were color coded according to the grain misorientations of the (001) <100> orientation in the maps legend. Figure 2a shows the results of OIM for sample 1. The vast majority of Y_2_O_3_ grains had the misorientation angles with the sample normally concentrated in 2°~4°. Few grains showed green in the OIM image, which indicated that a small part of grain misorientation angles were distributed in 5°~7°. Figure 2b exhibits the OIM pattern for sample 2. The color distributions of the pattern were blue-green, green, and yellow-green, according the maps legend, the grain misorientation angles of Y_2_O_3_ were 5°~9°. Additionally, the percentages of the orientated grains within misorientation angles of less than 20° were 97 and 98 % in samples 1 and 2, respectively. Figure 3 shows (100), (110), and (111) pole figures of samples 1 and 2, which calculated by OIM from the EBSD measured data. Both samples showed pure and sharp (001) <100> cubic texture, while the poles of sample 1 in Fig. 3a were smaller than sample 2 in Fig. 3b. These results demonstrated that the biaxial texture of sample 1 was better than that of sample 2.

**Fig. 4:**
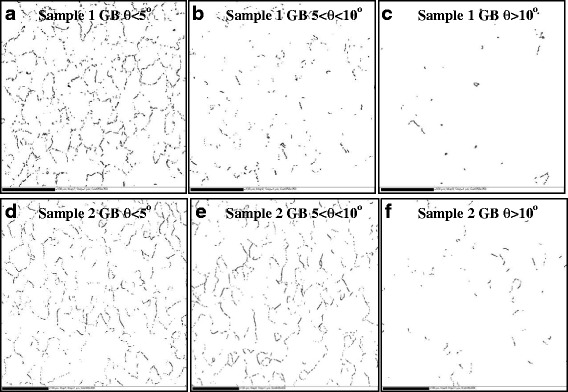
Total GB misorientation angles superimposed on the micrograph: **a**–**c** sample 1, **d**–**f** sample 2

Figure 4 shows GBs mapping of Y_2_O_3_ seed layer superimposed on the background of the grain image for samples 1 and 2, respectively. The GBs were divided into the varied misorientation angles: *θ* < 5°, 5° < *θ* < 10°, and *θ* > 10°. In sample 1, the GBs were almost completely concentrated of *θ* with 2°~5°. In Fig. 4b, the GBs from 5°~10° were not connected, and the segments were distributed in the maps. Furthermore, quite few *θ* values were bigger than 10°, which was shown in Fig. 4c. In sample 2, the majority of GBs allocated in 2°~10°, shown in Fig. 4d, e. And GBs with bigger than 10° were also very few. It can be seen that numerous low-angle GBs (*θ* < 10°) were present, while only a few high angle boundaries were detected in two samples. However, the low-angle GB distributions of two samples were quite different.

**Fig. 5:**
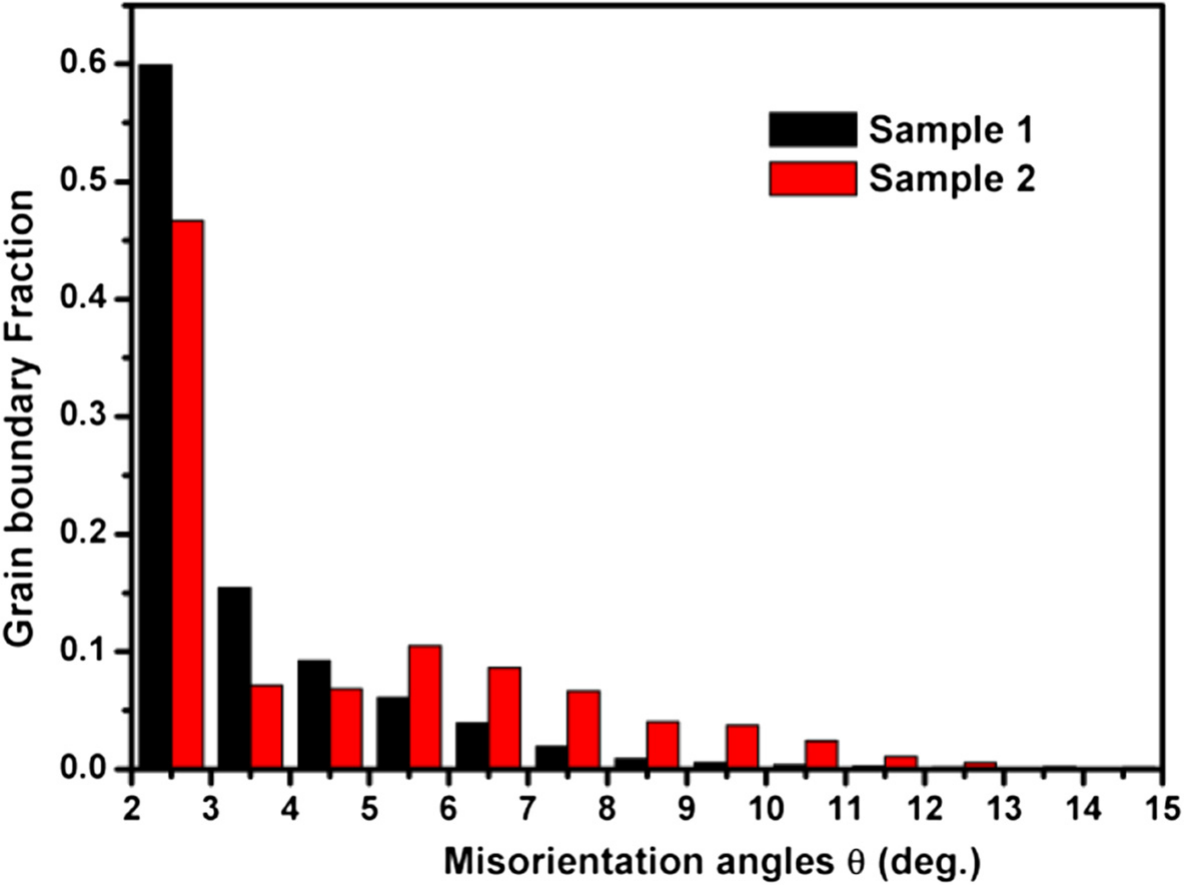
Fraction of total GB misorientation angle *θ* in each angular range for the samples 1 and 2

Figure 5 presented the quantified total GB misorientation angles *θ* distributed in the range from 2° to 15° for sample 1 and sample 2. There was a significant number of higher *θ* in sample 2, with more than 39 % greater than 6° and more than 45 % greater than 3°. The sample distribution exhibited a completely different shape, peaking in the 2°~3° and decreasing monotonically. In sample 1, the *θ* in the range of 6°~15° accounting for the proportion of the whole range was 15 %, and the *θ* in the range of 2°~5° was more than 85 %.

In order to verify the impact of different grain boundary networks on *J*_c_, the subsequent YSZ and CeO_2_ layer were deposited on the two samples by RF sputtering, and 1-μm-thick YBCO films were deposited by PLD. The *J*_c_ values of YBCO deposited on samples 1 and 2 were 1.58 MA/cm^2^ and 0.71 MA/cm^2^, respectively. Traditionally, GBs of YBCO can be described in two dimensions, and the PLD-derived YBCO films exhibited a perfect epitaxy on the buffer layers. There was no change in the textures and GB networks of YBCO and buffer layers. Considering that the surface morphology of YBCO films were not very dense, the grain boundary networks of YBCO were too difficult to measure by EBSD. And hence, we assumed the grain boundary networks of the buffer layer were completely transferred to YBCO films.

In the studies of YBCO deposited on bicrystal structures [28], the relationship between *J*_c_ and *θ* was used:

1 $$ {J}_{\mathrm{c}}\left(\theta \right)={J}_{\mathrm{c}}(0) \exp \left(-\theta /\alpha \right) $$

Here, *J*_c_(0) is the *J*_c_ values when *θ* is zero and the factor *α* is the relationship depending on the measured temperature and magnetic fields. For our experiment, the temperature is 77 K and the magnetic field is self field. Hence, *α* is set at 3.4, reported in the previous literature [29]. Due to the slight change of the true in-plane (100) texture between buffer layers and YBCO films, the out-of-plane GB misorientation angles are considered in the current study, which are a combination of [100] tilt and [100] twist, for a detailed description of the EBSD Euler angle (*φ*1, Φ, *φ*2) transfer in the in-plane ([001] tilt) and out-of-plane ([100] tilt and [100] twist) misorientation [30].

Equation (1) is the current flow transport behavior in one grain boundary with certain misorientation angle *θ*. For RABiTS process, there are thousands of GBs in films, as referred to the GB mapping superimposed in Fig. 4. Calculation of the relationship of all the GB misorientation angles *θ* and *J*_c_ values is very difficult. A simple model was proposed to investigate the effect of the grain boundary networks on the current transport. The EBSD data of two samples were handled by MATLAB software, and the data was incorporated into a hexagonal percolation model to calculate the *J*_c_/*J*_c_(0) of two samples.

**Fig. 6:**
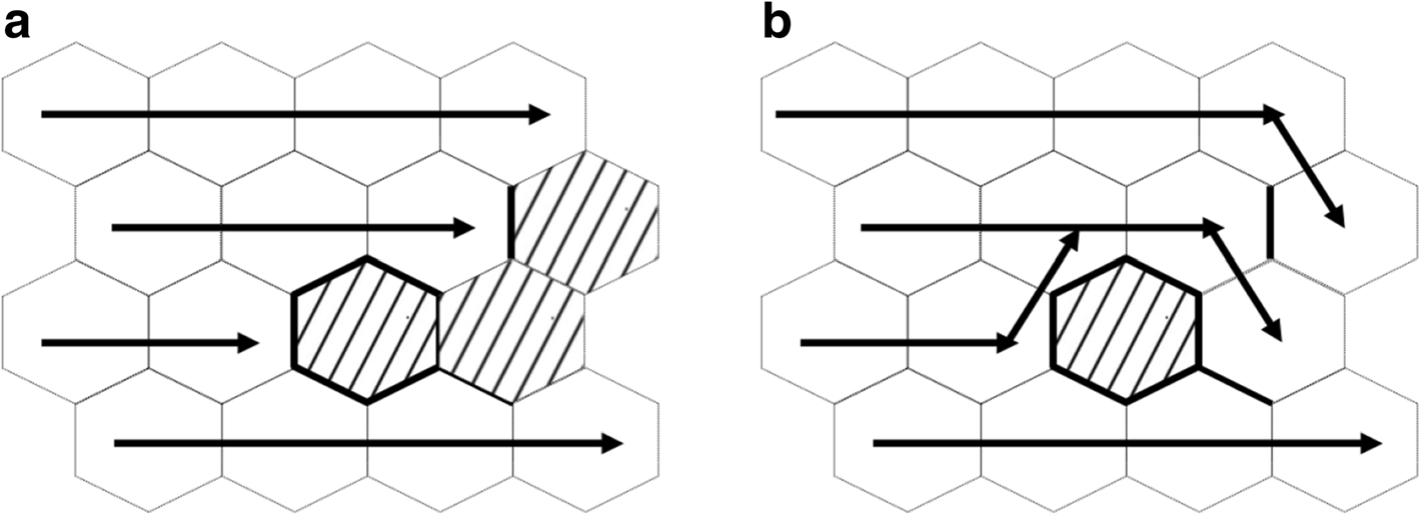
Two possible model parameters. **a** Percolation probability, **b** area percolated

The model is based on the percolation viewpoint, which is the current flow transport to the number of neighbor grains throwing the GBs. Figure 6 shows two possible model structures. The first is the percolation probability, which is probability of end to end percolation through the sample at a given threshold angle (*θ*_c_). Based on the threshold angle *θ*_c_, the GBs become either open or closed, while only the [100] tilt orientation grain boundaries are calculated. The second one is the area percolated, and the out-of-plane ([100] tilt and [100] twist) GB misorientation angles *θ* are calculated. If it is not possible for the percolation from end to end in the current transport direction, the percolation will be connected to the neighbor grains at a given threshold angle *θ*_c_. If percolation occurs, this model parameter calculating the fraction of the hexagonal point is accessible.

**Fig. 7:**
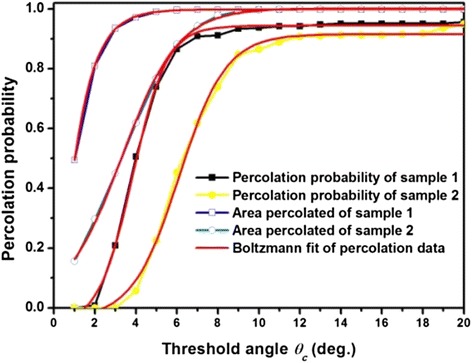
Percolation probability and area percolated of the two samples, the percolation probability data are indicated by *open symbols*, and the area percolated is indicated by *full symbols*

A fitting procedure program, written by MATLAB, models the GB misorientation angle *θ* distribution. The threshold angle *θ*_c_ is defined from 1° to 20°, and for any threshold angle *θ*_c_, the GB misorientation angles *θ* of two samples are handled in two possible percolation models, which are plotted in Fig. 7. The solid symbols are the percolation probability, and the open symbols are the area percolated values. The curves for the possible percolation of two samples are steep. The percolation probability of sample 1 with a relatively sharp transition is between 2° and 5°. However, the transition of sample 2 is between 3° and 10°. For the model of area percolated, the transition of two samples is 1°~4° and 1°~8°, respectively. For sample 1, the curves show a sharp transition and ranges are smaller than 5°. The transition of sample 2 is broader than sample 1, which is relative to the Fig. 4 results.

The full curves in Fig. 7 showed the percolation data was fitted by Boltzmann function. The form of the Boltzmann function is as follows

2 $$ f(x)={\mathrm{k}}_2+\left({\mathrm{k}}_1-{\mathrm{k}}_2\right)/\left(1+ \exp \left(\left(\mathrm{x}-\xi \right)/\delta \right)\right) $$

*J*_c_/*J*_c_(0) was calculated through the percolation data by Eqs. (1) and (2):

3 $$ {J}_c/{J}_c(0)={\displaystyle \int f(x){J}_c(x)}dx $$

Table 1 shows the results calculated by Eq. (3). For the model of percolation probability, the *J*_c_/*J*_c_(0) of samples 1 and 2 were only 0.347 and 0.186, respectively. And for the model of area percolated, the *J*_c_/*J*_c_(0) of the two samples were 0.898 and 0.361, respectively. Due to significant difference in grain boundary misorientation angle *θ* distribution of two samples, there was a significant disparity in the *J*_c_ results of the model calculated. Plainly, the model of the area percolated was closed to the true current transport mode and true *J*_c_ results in Table 1. As the vast majority of GB misorientation angles *θ* distributed in smaller than 5°, the *J*_c_ values could reach 90 % of *J*_c_(0).

**Table 1:** The results of the two models, which were calculated by Eq. (3)

	Percolation probability	Area percolated	*J*_c_ of YBCO
Sample 1	*J*_c_/*J*_c_(0) = 0.347	*J*_c_/*J*_c_(0) = 0.898	1.58 MA/cm^2^
Sample 2	*J*_c_/*J*_c_(0) = 0.186	*J*_c_/*J*_c_(0) = 0.361	0.71 MA/cm^2^
*J*_c_ sample 1: *J*_c_ sample 2	1.87	2.49	2.23

## Conclusions

We investigated the significant differences in *J*_c_ values of GB misorientation angles *θ*. For a high-quality buffer layer, the improved alignment of the out-of-plane texture and GB misorientation angles were indicated by XRD and EBSD. A simple percolation model was used to simulate the current flow in different samples. The enhancement of the current carry capacity of the YBCO films was attributed to GB misorientation angles *θ* of the buffer layer.
